# Investigation of the Role of TNF-α Converting Enzyme (TACE) in the Inhibition of Cell Surface and Soluble TNF-α Production by Acute Ethanol Exposure

**DOI:** 10.1371/journal.pone.0029890

**Published:** 2012-02-03

**Authors:** Kristine von Maltzan, Wei Tan, Stephen B. Pruett

**Affiliations:** 1 Department of Cellular Biology and Anatomy, Louisiana State University Health Sciences Center, Shreveport, Louisiana, United States of America; 2 Department of Basic Sciences, College of Veterinary Medicine, Mississippi State University, Mississippi State, Mississippi, United States of America; Louisiana State University, United States of America

## Abstract

Toll-like receptors (TLRs) play a fundamental role in the immune system by detecting pathogen associated molecular patterns (PAMPs) to sense host infection. Ethanol at doses relevant for humans inhibits the pathogen induced cytokine response mediated through TLRs. The current study was designed to investigate the mechanisms of this effect by determining whether ethanol inhibits TLR3 and TLR4 mediated TNF-α secretion through inhibition of transcription factor activation or post-transcriptional effects. In NF-κB reporter mice, activation of NF-κB in vivo by LPS was inhibited by ethanol (LPS alone yielded 170,000±35,300 arbitrary units of light emission; LPS plus ethanol yielded 56,120±16880, p = 0.04). Inhibition of protein synthesis by cycloheximide revealed that poly I:C- or LPS-induced secreted TNF-α is synthesized de novo, not released from cellular stores. Using real time RT-PCR, we found inhibition of LPS and poly I:C induced TNF-α gene transcription by ethanol. Using an inhibitor of tumor necrosis factor alpha converting enzyme (TACE), we found that shedding caused by TACE is a prerequisite for TNF-α release after pathogen challenge. Flow cytometry was used to investigate if ethanol decreases TNF-α secretion by inhibition of TACE. In cells treated with LPS, ethanol decreased both TNF-α cell surface expression and secretion. For example, 4.69±0.60% of untreated cells were positive for cell surface TNF-α, LPS increased this to 25.18±0.85%, which was inhibited by ethanol (86.8 mM) to 14.29±0.39% and increased by a TACE inhibitor to 57.88±0.62%. In contrast, cells treated with poly I:C had decreased secretion of TNF-α but not cell surface expression. There was some evidence for inhibition of TACE by ethanol in the case of LPS, but decreased TNF-α gene expression seems to be the major mechanism of ethanol action in this system.

## Introduction

We and others have reported that ethanol, at concentrations relevant to human exposure, inhibits signaling through toll-like receptors [1], [2], [3], [4] and the resultant production of a wide range of cytokines and chemokines [5]. However, it has also been reported that ethanol administration suppresses soluble TNF-α production at the level of translation or release of the protein [6], [7]. This would imply that either TNF-α mRNA or TNF-α protein is being stored in the cell, and available at the time of pathogen challenge and that ethanol acts by inhibiting release rather than production of the mRNA or protein. The release of TNF-α from the cell membrane is dependent on TNF-α converting enzyme TACE, and it has been reported that the function of this enzyme at the cell surface is inhibited by ethanol [6], [7]. It is known (and our results confirm) that macrophages express TNF-α in the cell membrane even when they are not activated, but it is not clear if the amount is sufficient to account for a substantial portion of the soluble TNF-α produced by these cells upon stimulation. This laboratory has reported that ethanol inhibits TLR4 mediated signaling and subsequent NF-κB activation and cytokine production. Thus, there seems to be evidence supporting TACE as well as TLR4 as targets of immunosuppressive effects of ethanol. The study described here was designed to investigate the relative role of these two mechanisms in the same experimental system.

The study described here focuses on TLR3 and TLR4. The TLR4 molecule senses lipopolysaccarides (LPS) from the outer membrane of gram-negative bacteria. The natural ligand for TLR3 is double stranded RNA (dsRNA) of viral origin. It also senses the synthetic dsRNA analog polyinosinic:polycytidylic acid (poly I:C). All TLRs apart from TLR3 recruit the adapter protein MyD88 (myeloid differentiation primary response gene 88) to the TIR domain [8], [9], leading to signaling through NF-κB or MAPK to induce transcription of inflammatory cytokine genes. However, TLR3 signaling depends on the adapter protein TRIF (TIR-containing adaptor inducing IFN-β), [10], [11], to activate a pathway that results in activation of the transcription factor IRF-3 (IFN-regulatory factor 3) [12] to induce type I interferons. TLR3 can also activate NF-κB or the MAPK pathway by mechanisms that are not fully understood. Because TLR4 can recruit both adapter proteins, MyD88 and TRIF [10], there is a signaling pathway that is common to TLR3 and TLR4.

Tumor necrosis factor alpha (TNF-α) is a proinflammatory cytokine and a major player in the regulation of the immune response. It is mainly produced by macrophages and other cells of the immune system upon pathogen challenge. Pro-TNF-α is a homotrimeric type II transmembrane protein [13], that is proteolytically cleaved from the cell surface by TACE [14], [15]. TACE, also known as A Disintegrin And Metalloproteinase 17 (ADAM17), is a type I transmembrane protein that is constitutively expressed. Its mRNA has been found in most tissues [14]. Human TACE cleaves the protein bond of pro-TNF-α between the amino acids Ala-76 and Val-77 [14]. Mouse TACE cleaves between Thr-79 and Leu-80 of the mouse precursor TNF-α cleavage site [16]. Catalytic activity of TACE is induced by LPS [17], [18], and downregulated by IL-10 in an early stage, and by Tissue Inhibitor of Metalloproteinases-3 (TIMP-3) in a later stage. The mechanism of TACE activation is still poorly understood. It has been reported, that phosphorylation of TACE at Thr-735 leads to activation and protein trafficking [19], [20], [21]. TACE is inhibited by a range of synthetic matrix metalloproteinase (MMP) inhibitors. Its natural inhibitor is TIMP-3 [22]. The synthetic metalloproteinase inhibitor, TNF-α processing inhibitor-0 (TAPI-0) has been widely used in vitro [23], [24].

Inhibition of TACE and inhibition of signaling by ethanol could both contribute to the inhibition of the release of soluble TNF-α. The study described here was designed to evaluate both mechanisms in the same experimental system. The major novel finding of this study was that inhibition of TACE by ethanol was not the major mechanism that decreased production of soluble TNF-α. In particular, we found that ethanol decreased both cell surface and released TNF-α, unlike a known TACE inhibitor, which increased cell surface TNF-α but decreased the released form. This suggest that inhibition of signaling and TNF-α gene expression (which were confirmed here) was a more important mechanism of action in this experimental system.

## Results

### New protein synthesis is required for maximum production of secreted TNF-alpha

The protein synthesis inhibitor cycloheximide interferes with the translocation step at the ribosome, thereby blocking translational elongation. To test if TNF-α is available from cellular stores, synthesis of new protein was inhibited by cycloheximide. If TNF-α protein were available in the cell, blocking protein synthesis would not significantly affect the amount of TNF-α measured in cell culture supernatants after challenge with a TLR ligand.

**Figure 1:**
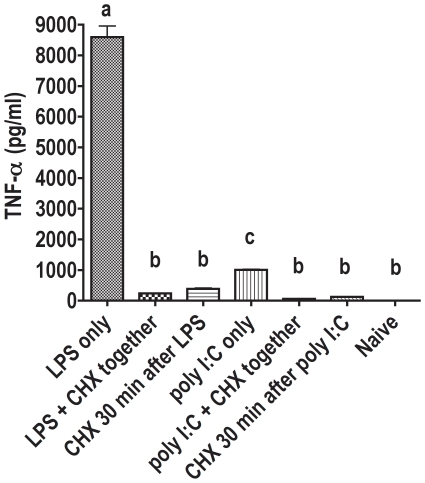
Mouse TNF-α ELISA assay from cell culture supernatants. Each group contained 6 samples. Cells were treated with LPS/poly I:C alone, with cycloheximide and LPS/poly I:C at the same time point, or with LPS/poly I:C first and cycloheximide 30 min later. Naive, untreated cells served as control. Bars with no shared letters are significantly different (p<0.05).

The effects of LPS or poly I:C alone and in combination with cycloheximide were examined. Cycloheximinde was added to cultures of RAW264.7 cells either at the same time point as LPS or poly I:C, or 30 min after the addition of LPS or poly I:C to the cell culture (Figure 1). Two hours after LPS or poly I:C addition, cell culture supernatants were harvested, and ELISA was performed.

Added to the cell culture supernatant as long as 30 min after LPS or poly I:C, cycloheximide caused a major decrease in TNF-α, verified by ELISA. Cycloheximide almost completely abrogated TNF-α synthesis after challenge with TLR ligands. As a control, cycloheximide was also incubated with the TNF-α ELISA standard, and it was shown not to interfere with the ELISA system. From these findings it can be inferred that de novo synthesis of TNF-α happens after pathogen challenge, and that the TNF-α on the cell membrane does not represent a sufficient “storage” form of TNF-α, which is ready for secretion upon stimulation. Therefore, the mechanism by which ethanol decreases TNF-α secretion after pathogen challenge must include effects other than or in addition to preventing its release from storage. Ethanol could act at the transcriptional or post-transcriptional level.

### Inhibition of TNF-α secretion starts at the signaling pathway

**Figure 2:**
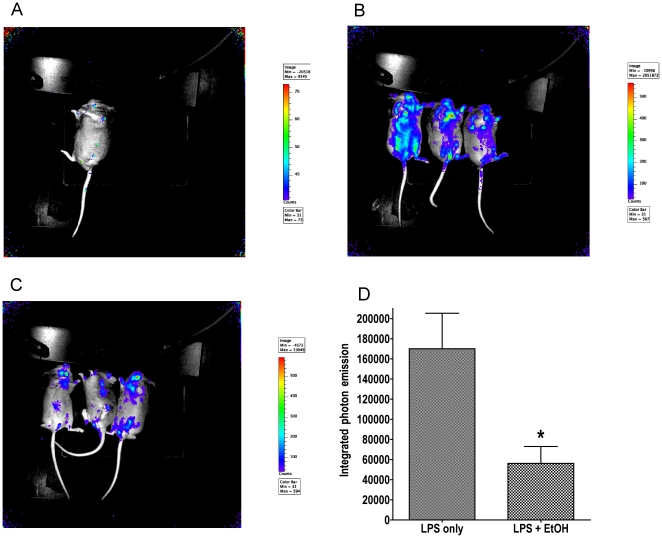
Comparison of NF-κB expression in reporter mice treated with LPS alone or ethanol and LPS. **A** For the naive mouse, luminescence was 1.627×10^4^ CCD camera counts. **B** In the group treated with LPS only, 2.406×10^5^ counts were measured for mouse one, 1.347×10^5^ counts for mouse two, and 8.485×10^4^ counts for mouse three. **C** In the group treated with LPS+86.8 mM EtOH, 4.754×10^4^ counts were measured for mouse one, 3.213×10^4^ for mouse two, and 8.869×10^4^ for mouse three. **D** Mice treated with 6 g/kg ethanol before LPS showed significant reduction of luminescence (* = LPS plus EtOH vs. LPS only: P<0.05).

To investigate if ethanol suppresses NF-κB signaling in vivo, we looked at the effect of LPS alone or in combination with ethanol in transgenic mice. The NF-κB reporter mice carry the luciferase gene driven by an NF- κB responsive promoter. Challenge with TLR ligands activates the NF- κB dependent pathway causing expression of luciferase in the reporter mice which is then detected by injecting luciferin, resulting in light emission in tissues with high NF-κB activation. One group of mice was gavaged with ethanol 5 min before LPS injection, and one group was only injected with LPS. A control mouse did not receive either ethanol or LPS. After LPS injection (1.5 hr, the time of near maximal luciferase activity), light emission was measured with the IVIS imaging system (Figure 2).

Mice treated with LPS show significantly more NF-κB activation than mice treated with EtOH+LPS, measured by overall light emission in each mouse (P<0.05). These findings corroborate the hypothesis, that EtOH inhibition is effective at the level of NF-κB dependent signaling, and is not limited to post-transcriptional effects.

### Ethanol inhibits LPS- and poly I:C induced TNF-α gene transcription

**Figure 3:**
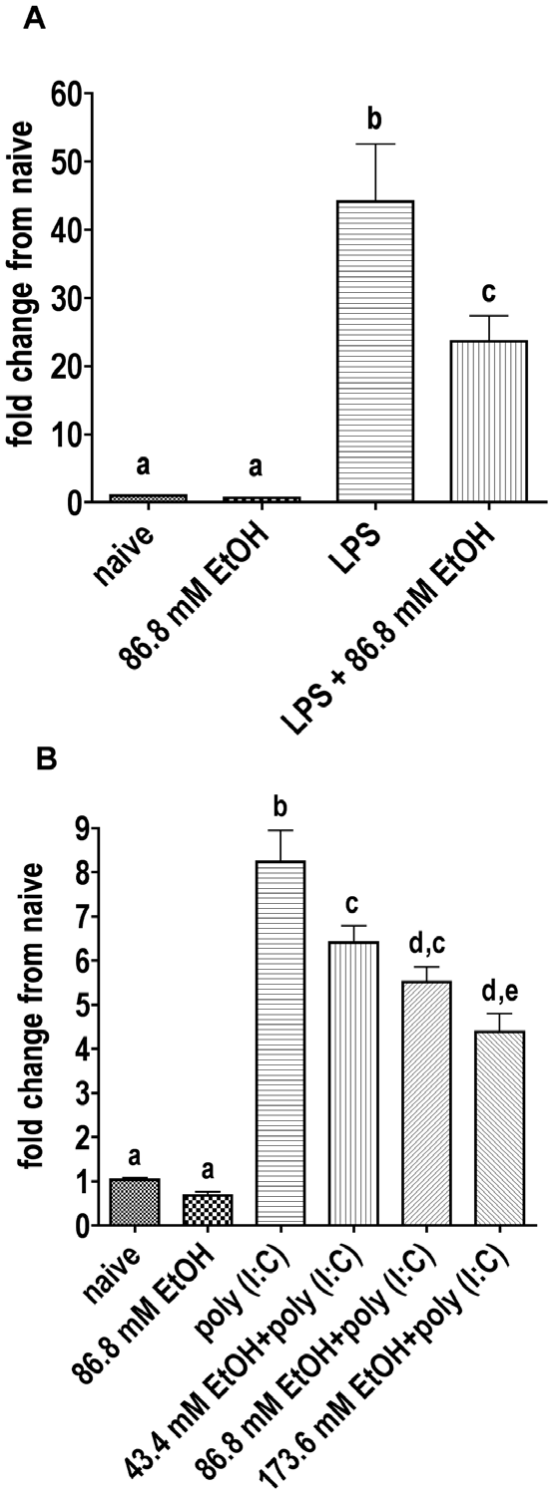
Real time RT-PCR with mRNA isolated from RAW264.7 cells treated with ethanol and LPS or poly I:C. Naive groups received no treatment. **A** Appropriate groups were treated with ethanol for 30 min, then with LPS for further two hours. The average of two experiments, each with three replicates, were pooled. Each sample replicate was doubled in the PCR plate. 86.8 mM ethanol significantly reduced the LPS induced TNF-α mRNA expression (P<0.001). Treatment with ethanol only was not significantly different from naive. **B** Appropriate groups were treated with different concentrations of ethanol for 30 min, then with poly I:C for further two hours. The average of two experiments, one with three replicates, and one with two replicates, is shown. Each sample replicate was doubled in the PCR plate. A concentration of 43.4 mM EtOH and higher significantly reduced the poly I:C induced TNF-α mRNA expression (P<0.01). Ethanol without poly I:C was not significantly different from naive. Results were normalized to 18S and analyzed using the ΔΔCt method. Bars with no shared letters are significantly different (p<0.05).

A decrease in NF-κB activation by ethanol should be reflected by decreased cytokine gene transcription. Therefore, the effect of ethanol on the LPS- and poly I:C-induced TNF-α mRNA expression was evaluated by real time RT-PCR in RAW264.7 cells. Appropriate groups were incubated with ethanol for 30 min, and then with LPS or poly I:C for further two hours. Untreated naive cells served as control. RNA was isolated from cell lysates, and real time RT-PCR was preformed (Figure 3).

A concentration of 43.4 mM EtOH and higher in the poly I:C group, and 86.8 mM in the LPS group, significantly inhibited the pathogen induced TNF-α mRNA expression in RAW264.7 cells (P<0.01). It should be noted that the dosage of ethanol used in the NF-κB reporter mice (6 g/kg) yields a peak blood ethanol concentration of ∼86 mM [25], so results from inhibition of NF-κB activation in vivo and inhibition of TNF-α production in vitro with ethanol at 86.8 mM reflect similar exposure. As noted in our previous publications, 86.8 mM would represent the high end of the concentration range that can be found in humans, but such concentrations are not as rare as might be expected [26], [27]. In addition, mice clear ethanol more rapidly than humans, so obtaining similar area under the concentration vs. time curve in mice as reported in humans requires a higher dosage and greater peak blood ethanol concentration in mice. For example, in humans with a mean blood ethanol concentration of 299 mg/dL, the clearance rate was observed to be 20.4 mg/dL/hr [28]. Our results with the mouse model used in the present study indicate that mice with virtually the same initial blood ethanol concentration have a clearance rate of 37.18 mg/dL/hr [25] (using a dosage of 5 g/kg). Finally, we have recently shown that TNF-α production induced by *Escherichia coli* in mice is almost eliminated by ethanol at both 4 g/kg and 6 g/kg in mice [5], and a dosage of 4 g/kg in our model yields a peak blood ethanol concentration of ∼43 mM (200 mg/dL) [25], a concentration frequently observed in binge drinkers. In the poly I:C group, the concentration of 173.6 mM EtOH was added to show the direction of the EtOH effect, although this concentration is not relevant in humans. The greater induction of TNF-α mRNA by LPS than poly I:C (Figure 3) is consistent with the greater induction of TNF-α protein by LPS than poly I:C (Figure 1). These results support the idea that ethanol affects transcription of TNF-α in activated macrophages.

### Secretion of TNF-α is dependent on TACE in cells activated with LPS or poly I:C

**Figure 4:**
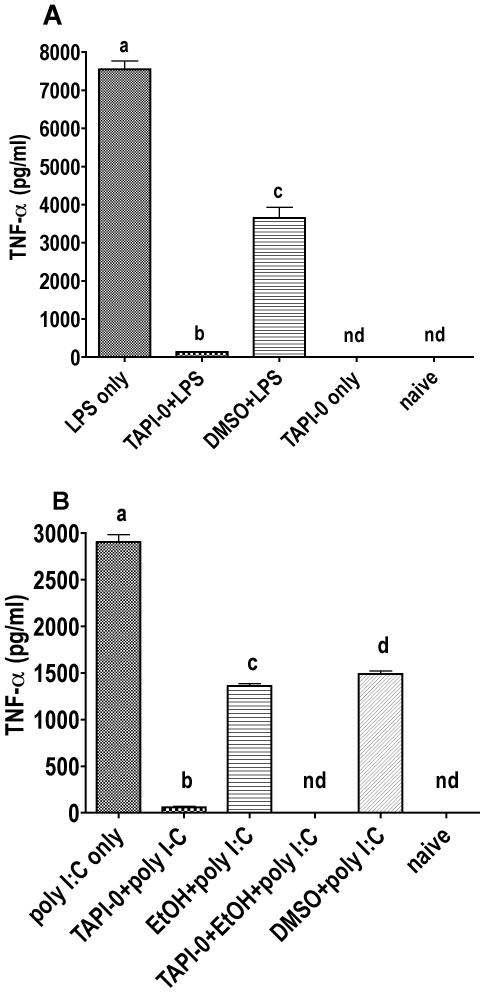
Effect of TACE inhibition on the LPS and poly I:C induced TNF-α response in RAW264.7 cells. TNF-α levels were measured by ELISA from cell culture medium immediately after collection. Each group contained 6 samples. Naive groups received no treatment. **A** Cells were treated with 25 µg/ml TAPI-0, 5 µl/ml DMSO, and/or 100 ng/ml LPS. Treatments were given at the same time point and cells were incubated 2 h. **B** Appropriate groups were treated with 86.8 mM EtOH and incubated 30 min. Cells were treated with 25 µg/ml TAPI-0, 5 µl DMSO, and/or 50 µg/ml poly I:C, and incubated further 2 h. Bars with no shared letters are significantly different (p<0.05; nd = not detectable).

Tumor necrosis factor alpha converting enzyme (TACE/ADAM17), a metalloprotease, is located in the cell membrane and cleaves pro-TNF-α from the cell surface. TAPI-0 (TNF-α processing inhibitor-0) is an inhibitor of metalloproteases. In this experiment, the extent of TACE contribution to the level of TNF-α shed into the cell culture supernatant was evaluated (Figure 4). In the LPS experiment, RAW264.7 cells were treated with 10 ng/ml LPS alone, LPS plus 25 µg/ml TAPI-0, LPS plus 5 µl DMSO (control for the DMSO used to dissolve TAPI-0), or TAPI-0 alone. In the poly I:C experiment, RAW264.7 cells were treated with 50 µg/ml poly I:C alone, 25 µg/ml TAPI-0 plus poly I:C, 86.8 mM EtOH plus poly I:C, TAPI-0 plus 86.8 mM EtOH plus poly I:C, or 5 µl DMSO. In both experiments, naive untreated cells served as control. TAPI-0 was dissolved in DMSO. A control group was included to account for any inhibition of TNF-α caused by DMSO. First, TNF-α secretion was compared between cells treated with TAPI-0 plus LPS, LPS alone, or DMSO plus LPS (Figure 4A). It was obvious, that DMSO itself caused a significant decrease in TNF-α (LPS only vs. DMSO plus LPS: P<0.001). However, when the groups TAPI-0 plus LPS and DMSO plus LPS were compared, a highly significant decrease in TNF-α due to TACE inhibition was seen (P<0.001). In the naive group and the group treated only with TAPI-0, no TNF-α could be detected by ELISA. Next, the effect of TAPI-0 on cells activated with poly I:C was examined. Poly I:C alone and DMSO plus poly I:C were compared to TAPI-0 plus poly I:C, 86.8 mM EtOH plus poly I:C, or TAPI-0 plus 86.8 mM EtOH plus poly I:C. Naive, untreated cells served as control (Figure 4B). Ethanol significantly inhibited the poly I:C induced TNF-α response (P<0.001). Compared to DMSO plus poly I:C, TAPI-0 significantly reduced TNF-α in the cell culture supernatant (P<0.001). In the group treated with EtOH before adding TAPI-0 and poly I:C, no TNF-α was detected by ELISA. In the naive group, TNF-α level was below the limit of detection. These results indicate that secreted TNF-α measured by ELISA in cell culture supernatants is mainly, if not entirely, due to cleavage by TACE.

### Inhibition of TACE increases, and ethanol decreases, cell surface expression of TNF-α in LPS treated macrophage-like cells

**Figure 5:**
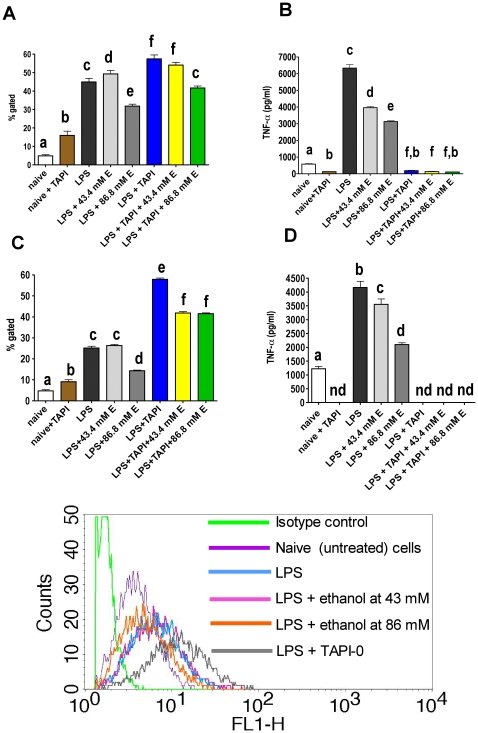
Surface expression of TNF-α on RAW264.7 cells measured by flow cytometry, and TNF-α ELISA from cell culture supernatants of the same experiments. Results shown in **A** and **B** were obtained in one experiment and results shown in **C** and **D** were obtained in an independent experiment. In some groups, no TNF-α was detected (nd). Each group contained 5 samples. Two repeat experiments are shown. Cells were treated with ethanol (either 43.4 mM or 86.8 mM), 100 ng/ml LPS or 20 µg/ml TAPI-0, or a combination of these treatments. ELISA was performed from cell culture supernatants of each group. The percentage of gated cells (positive for TNF-α surface expression by flow cytometry) for each group is shown in **A** & **C**. The ELISA results from cell culture supernatants of the cells used for flow cytometry are depicted in **B** and **D**. Bars designated by the same letter are not significantly different (p>0.05); bars with no shared letters are significantly different (p<0.05). Histograms for representative samples from key groups for the experiment shown in A are shown below panels **C** and **D**.

The previous experiments suggest that ethanol decreases signaling through NF-κB and TNF-α synthesis, and therefore it should decrease surface TNF-α. If ethanol also decreases TACE activation, an increase in surface TNF-alpha could be expected because of a diminished rate of shedding. As an indirect approach to evaluate the effect of ethanol on TACE activity, and to quantitate surface TNF-α with and without ethanol, the expression of surface TNF-α was measured by flow cytometry. TACE was inhibited by TAPI-0 to examine the effect of ethanol on TNF-α surface expression before shedding. For all flow cytometry experiments, TAPI-0 was dissolved in RPMI to avoid the cytotoxic effect of DMSO. As noted in Materials and Methods, DMSO was used at the recommendation on the manufacturer, but at the relatively low concentrations of TAPI-0 needed for this study, the compound was soluble in an aqueous solution without DMSO. The RAW264.7 cells were transferred into microcentrifuge tubes at 2.5×10^6^/ml. Appropriate groups were treated with 43.4 and 86.8 mM ethanol for 30 min, and then with 100 ng/ml LPS and/or 20 µg/ml TAPI-0 for further 2 h. Supernatants were collected and subjected to ELISA (Figure 5 B and D). Cells were labeled with monoclonal rat anti mouse TNF-α FITC conjugate and flow cytometry was performed (Figure 5A and C). Naive unlabeled cells showed very low fluorescence. Labeling with isotype control was performed with LPS treated cells in each experiment, and fluorescence was similar to naive unlabeled cells (data not shown). The results are shown as % gated (Figure 5A and C). The gate was set to exclude ∼99% of the cells in isotype or unlabeled controls and to exclude forward scatter values indicating that cell clusters rather than single cells were being assessed. Results for mean fluorescence intensity were also calculated, but the pattern of effects was very similar to that for % gated, so only the results for % gated are shown here. In the LPS treated group, TNF-α surface expression was significantly above naive (P<0.001 for % gated in both experiments). Analysis by ELISA showed a significant increase in TNF-α in the supernatant versus naive (P<0.001 in both experiments). Whilst high surface expression of TNF-α reflects an increase in gene transcription and/or decreased TACE activity, high TNF-α in the cell culture supernatant is due to TACE activity in the activated macrophage. Treatment with 86.8 mM ethanol significantly inhibited LPS induced TNF-α surface expression versus LPS only in both experiments (P<0.001 for % gated). TNF-α in cell culture supernatants measured by ELISA was decreased accordingly (P<0.001), reflecting a decrease in TNF-α synthesis. A decrease in TACE activity could have potentially contributed to a decreased TNF-α release by macrophages. However, a decrease in surface TNF-α caused by 86.8 mM ethanol indicates that decreased TNF-α release is not caused solely by a decrease in TACE activity, which would yield an increase in surface TNF-α.

### Ethanol decreases TNF-α secretion, but not TNF-α cell surface expression in poly I:C treated macrophage-like cells

**Figure 6:**
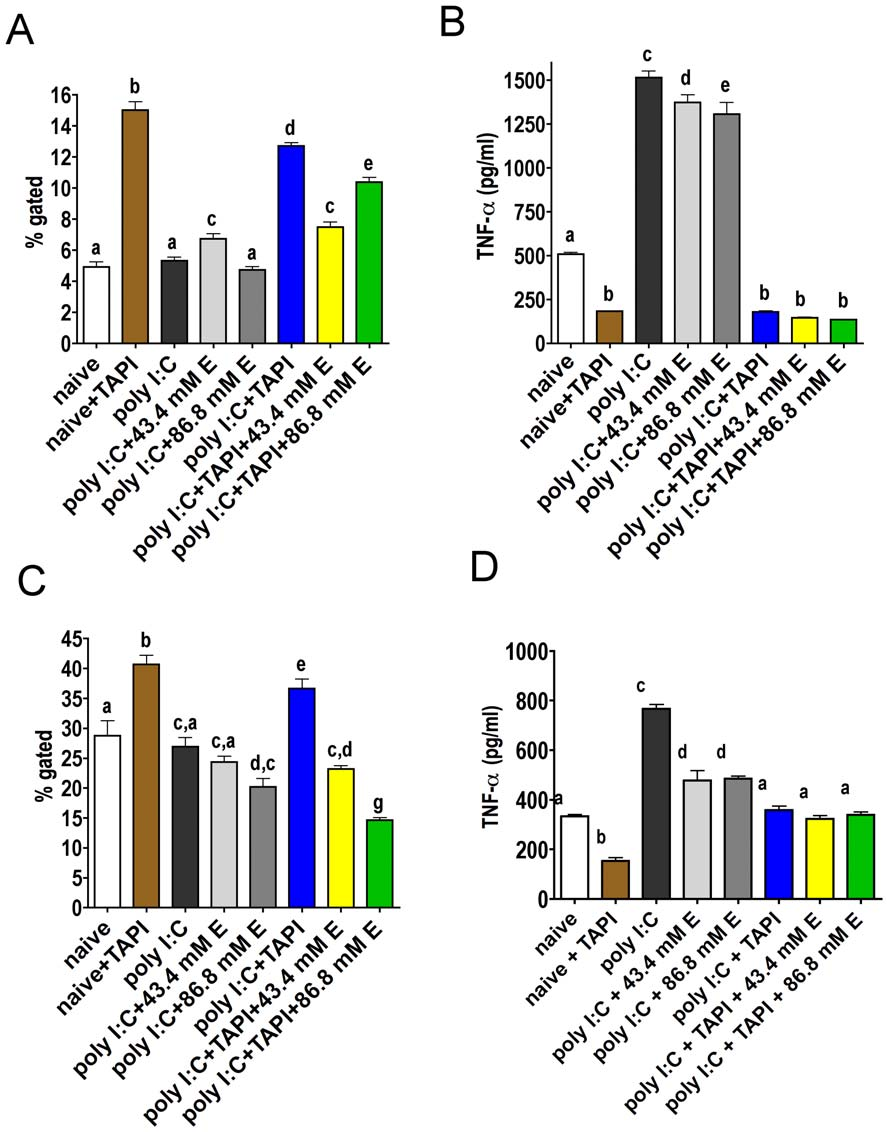
Surface expression of TNF-α in RAW264.7 cells measured by flow cytometry, and TNF-α ELISA from cell culture supernatants of the same experiments. Results shown in **A** and **B** were from one experiment and results shown in **C** and **D** are from an independent experiment. Each group contained 5 samples. Cells were treated with ethanol (either 43.4 mM or 86.8 mM, as indicated in the Figure), 50 µg/ml poly I:C, 20 µg/ml TAPI-0 (with no DMSO), or a combination of these treatments. ELISA was performed from cell culture supernatants of each group. The percentage of gated cells for each group is shown in **A** and **C**. Results for TNF-α ELISA from cell culture supernatants of the cells used for flow cytometry are depicted in **B** and **D**. Values for bars designated by the same letter are not significantly different (p>0.05); values for bars with no shared letters are significantly different (p<0.05).

The above described experiments were repeated with the TLR3 activator poly I:C. Inhibition of poly I:C induced TNF-α gene expression by ethanol, as suggested by the real time RT-PCR results, should be reflected by a decrease in TNF-α surface expression similar to the results with LPS. The RAW264.7 cells were treated and labeled as in the above experiments, with the exception that 50 µg/ml poly I:C was used instead of LPS. The fluorescence for naive unlabeled cells and isotype control with poly I:C treated cells were similar and with a mean fluorescence intensity less than 10 (data not shown). Two independent experiments were conducted (experiment 1 shown in 6 A and B; experiment 2 shown in 6 C and D). Surface TNF-α expression is indicated as % gated (Figures 6 A and C). An ELISA was performed from cell culture supernatants of the cells used for flow cytometry (Figures 6 B and D). Similar to the previous experiments, TACE inhibition caused a significant increase in surface TNF-α in naive cells (P<0.001 for % gated both experiments), and a significant decrease in TNF-α in the culture supernatant (P<0.001 for both experiments). In contrast results obtained with LPS, treatment with poly I:C did not cause a change in the amount of surface TNF-α in either experiment. However, as with LPS, the increase in secreted TNF-α detected by ELISA was significant (P<0.001 vs. naive for both experiments). If TNF-α surface expression is determined by rate of synthesis and insertion of TNF-α minus the rate of cleavage by TACE, the results reported here seem to indicate that increased TNF-α membrane insertion in poly I:C treated cells is precisely balanced by increased cleavage by TACE. As expected, TNF-α surface expression was significantly higher in the poly I:C plus TAPI-0 group than in the poly I:C only group (P<0.001 for % gated both experiments), and TNF-α detection in the supernatants was significantly lower (P<0.001 in both experiments). Treatment with 43.4 mM ethanol before poly I:C did not change the amount of TNF-α surface expression in either experiment, but it decreased TNF-α in cell culture supernatants (P<0.01 for both experiments). Treatment with 86.8 mM ethanol before poly I:C decreased TNF-α surface expression only in the second experiment (P<0.01 for % gated), but TNF-α in cell culture supernatants was significantly decreased in both experiments (P<0.001). The effect of ethanol on total TNF-α membrane insertion was revealed by inhibition of shedding. Groups treated with 43.4 mM or 86.8 mM ethanol before poly I:C and TAPI-0 showed a significant decrease in surface TNF-α versus poly I:C plus TAPI-0 (P<0.001 for % gated). It seems likely that this was due to a decrease in TNF-α production, because TAPI-0 almost completely blocked release of TNF-α, thus almost completely inhibited TACE. This left only alterning TNF-α production and eliminated alteration of TACE activity as the mechanism of this action of ethanol.

## Discussion

It has been widely accepted that excessive ethanol consumption increases susceptibility to infections, especially pneumonia. However, the mechanisms underlying the effects of alcohol on host defense are not fully understood. Studies in mouse models show that TNF-α plays a key role in host defense [29], [30], [31]. The mechanism of action of ethanol on TLR4 (LPS)-induced macrophage responses (including TNF-alpha production) is very similar in human and mouse cells [32].

A major reason for conducting this study were reports indicating that TNF-alpha production was inhibited by ethanol primarily at the post-transcriptional level [6], [7]. Our previous studies indicated inhibition at the level of transcription [2], but we had not previously evaluated the role of TACE and whether it could be a major molecular target of ethanol. In the study described here, we investigated whether the inhibition of LPS- or poly I:C-induced TNF-α production by concentrations of ethanol relevant in human binge drinking reflects inhibition of transcription or post-transcriptional events. The results support an important role for decreased transcription, but not for decreased TACE activity in the inhibition of TNF-alpha production by ethanol.

Zhang and colleagues [6] reported that acute ethanol exposure of a human and a murine cell line (Mono Mac 6 and DRM) decreased activation of TACE and release of TNF-α. Our results did not seem to fully coincide with theirs, but it should be noted that it remains possible that there was a decrease in TACE activity caused by ethanol in our experimental system, even though the evidence as a whole indicates inhibition of TACE function is not the major mechanism of ethanol action. These investigators also did not find decreased expression of TNF-α mRNA, suggesting that the overall experimental system differs from ours, possibly because of differences among the cell lines used.

To better understand the dynamics of TNF-α production and release of the soluble form of TNF-α, we inhibited protein synthesis with cycloheximide after challenge with TLR ligand. It was shown that TNF-α is primarily synthesized de novo after activation with LPS or poly I:C. Even though the cycloheximide experiment does not exclude that TNF-α protein is stored in the cells (in excess of the small amount found in the membrane), it is unlikely, because TNF-α synthesis was abrogated even when protein synthesis was inhibited 30 min after TLR activation.

Ethanol has been shown by western blot and electrophoretic mobility shift assay (EMSA) to inhibit LPS induced NF-κB activation in human monocytes [33]. In an experiment with NF-κB reporter mice we tested, if ethanol inhibition of LPS induced TNF-α secretion starts at the signaling pathway, and if total NF-κB activation in vivo was inhibited by ethanol. Through in vivo imaging of NF-κB activity, we could show, that ethanol significantly inhibited LPS-induced activation of NF-κB induced gene transcription. This is consistent with our previous studies indicating that ethanol inhibits TNF-α production in response to LPS by inhibiting the formation of a fully functional TLR4 receptor complex and inhibiting TLR4-induced signaling at early, intermediate, and late (NF-κB) stages [34], [35]. However, it is unclear whether most of the NF-κB activation in this system was mediated by the primary stimulus (LPS or poly I:C), or by a secondary stimulus (such as TNF-α). In addition, it would be useful to know, in which organs and cell types ethanol suppresses NF-κB activation. It is known that Kupffer cells of the liver represent the largest concentration of macrophages in any single anatomical location, and it is noted, that this region is among the most inhibited by ethanol. Additional evidence would be needed to confirm, that the liver is a major location of cytokine production and that it is inhibited by ethanol.

In real time RT-PCR studies we evaluated if NF-κB inhibition by ethanol leads to a decrease in TNF-α mRNA. We found that the upregulation of TNF-α mRNA by LPS and poly I:C was consistently decreased by ethanol. This is consistent with our recent results indicating suppression by ethanol of TLR signaling and the production of a wide range of inflammation-related cytokines and chemokines [1], [5].

TACE releases TNF-α from the cell surface by shedding [14]. To evaluate if secretion of TNF-α is entirely dependent on TACE in cells activated with LPS or poly I:C, we used the metalloprotease inhibitor TAPI-0. Our results show that TNF-α secretion is almost completely abrogated if TACE is inhibited. This indicates, that shedding through TACE is required for release of the vast majority of TNF-α from the cells used in these studies. The metalloprotease inhibitor TAPI-0 is widely used to study protein shedding from the cell surface. In studies reviewed, the solvent for TAPI-0 was not mentioned, and there was no description of vehicle controls, e.g. [36], [37]. According to the manufacturer's instructions, TAPI-0 can be reconstituted in DMSO, EtOH, and 10% acetyl hydroxide in EtOH. To decrease the cytotoxic effect of DMSO, we used only 10% of the recommended volume, resulting in a final concentration of 0.5% in cell culture. Even at this concentration, we saw a significant decrease in ELISA detection of TNF-α, and a decrease in TNF-α cell surface expression in flow cytometry due to DMSO alone. Even at a concentration of 0.005%, DMSO significantly inhibited cell surface expression of TNF-α measured by flow cytometry (data not shown). In an in vivo study with rats it has been observed, that using DMSO as a vehicle for a drug may exert effects due to DMSO alone [38]. Therefore, we used TAPI-0 in a solution in RPMI for further experiments.

We showed by flow cytometry that treatment with LPS, but not treatment with poly I:C significantly increased cell surface TNF-α. Corresponding ELISA, however, showed increased free TNF-α after treatment with both, LPS and poly I:C, possibly due to an increase in TACE activity as well as upregulation of production of TNF-α. An upregulation of TACE mRNA with a peak after 2 h, and increased TACE surface expression measured by flow cytometry in human alveolar macrophages by LPS and IFN-γ after 20 h was reported by Armstrong et al. This LPS-induced TACE expression was found to be downregulated by IL-10 [39]. Our results show that poly I:C does not increase TNF-α synthesis to the same extent as LPS (Figure 3). Consequently, TACE activity may be sufficient to prevent the increase in surface TNF-α in poly I:C-treated cells. Inhibition of TACE by TAPI-0 increased surface TNF-α in both LPS and poly I:C treated cells as well as in naive cells through inhibition of shedding. It has been suggested that ethanol decreases TNF-α by inhibition of TACE [40], [31], or by physically preventing protein –protein interaction between TNF-α and TACE [41]. If ethanol acts primarily as a TACE inhibitor, we would expect surface TNF-α to be increased (as shown in cells treated with TAPI-0 in Figures 5 and 6). Possible explanations for the inconsistency between our results and those of another group are discussed in a previous paragraph.

Our results indicate that inhibition of NF-κB signaling and decreased TNF-α mRNA expression by ethanol occurred and acted to decrease surface TNF-α. The ethanol effect on TNF-α surface expression was more prominent in LPS treated cells. The free TNF-α in corresponding cell culture supernatants was significantly decreased by 43.4 mM ethanol in both LPS and poly I:C treated cells, which may be partly due to some inhibition of TACE activity. The effect of ethanol on TNF-α surface expression in the absence (or near absence) of TACE activity was revealed when shedding was inhibited by TAPI-0. In LPS and poly I:C treated cells. Ethanol at only 43.4 mM significantly decreased TNF-α surface expression, supporting the PCR results showing decreased TNF-α synthesis. Although we cannot exclude TACE inhibition caused by ethanol, our results indicate that decreased TNF-α production has more effect than any decrease in TACE activity that ethanol may cause.

Taken together, in this study we have shown, that new protein synthesis is required for maximum production of secreted TNF-α, and that TNF-α is not stored in the cell in a form ready for secretion. Our results indicate that inhibition of LPS- or poly I:C-induced TNF-α production by ethanol starts at the signaling pathway and is not limited to post-transcriptional effects (such as cleavage by TACE). Decreased transcription, not just TACE inhibition, is involved in decreased TNF-α production, as indicated by comparing the changes of surface and secreted TNF-α in ethanol and TAPI-0 treated cultures. The above findings give new insights into the mechanisms of ethanol inhibition of TLR signaling. However, it is possible, that ethanol interferes with other proteins in the TLR signaling cascade.

## Materials and Methods

### Cell culture assays

The RAW264.7 macrophage like cell line was purchased from American Type Culture Collection (ATCC). The RAW264.7 cells were cultured in RPMI 1640 with L-glutamine (# 61870-036, Invitrogen, Carlsbad, CA, USA) supplemented with 10% FBS (# 10437-028, Invitrogen) and penicillin-streptomycin (# P4333, Sigma-Aldrich, Saint Louis, MO, USA). They were used before the 10th passage. Cells were grown in 75 cm^2^ tissue culture flasks (Sarstedt INC, Newton, NC 28658-0468, USA) at 37° C and 5% CO_2_.

Poly I:C (# tlrl-pic-5) was purchased from Invivogen (San Diego, CA). Ultra Pure lipopolysaccharide (LPS) from E. coli serotype O111:B4 was obtained from List Biological Laboratories (# 421, List Biological Laboratories, INC., Campbell, CA, USA). The protein synthesis inhibitor cycloheximide was purchased from Sigma Aldrich (# C1988, Sigma Aldrich, St. Louis, MO). The TACE inhibitor TAPI-0 was purchased from Peptides International (# INH-3850-PI, Peptides International, Inc., Louisville, KY, USA). For all cell culture assays, cells were seeded into NUNC 24-well plates (# 142475, Thermo Fisher, Rochester, NY, USA) at 1×10^6^/ml, 1 ml/well, and grown for 24 h before the experiment. Appropriate wells were treated with 86.8 mM ethanol and incubated 30 min. Then, 100 ng/ml LPS or 50 µg/ml poly I:C were added to appropriate wells, and incubated further 2 h. Supernatants were collected or cells harvested for further procedures that were performed within 30 min after harvesting. For the protein synthesis inhibitor experiment, cells were cultured as described above, and appropriate wells (6 samples per group) were treated with 5 µg/ml cycloheximide.

### Quantitation of cytokines in cell culture supernatants by ELISA

A mouse cytokine ELISA kit was obtained from BD Biosciences (San Jose, CA, USA; BD OptEIA Mouse TNF-α Mono/Mono # 555268). The assay was carried out according to the manufacturer's specifications. NUNC maxisorp 96 well ELISA plates were used (# 439454, Thermo Fisher, Rochester, NY, USA).

### NF-κB reporter mice

Transgenic mice of C57BL/6J X CBA/J genetic background carrying the luciferase gene driven by an NF- κB response element, as described by Carlsen et al. [42], were obtained from Xenogen (Alameda, CA). D-Luciferin was purchased from Caliper (# 122796, Hopkinton, MA, USA). Mice were treated with ethanol by gavage at 6 g/kg as a 32% solution in water 5 min before NF-κB activation. LPS was administered via tail vein injection at 60 µg per mouse. One and a half hr later, mice were anesthetized with isoflurane by inhalation, and 150 mg/kg luciferin was injected intraperitoneally. Mice were oriented similarly and the ventral surface was imaged for all mice. Imaging was performed 5–10 min later with an IVIS imager (Xenogen, Alameda, CA). Mice were maintained and used in accord with the NIH Guide for Care and Use of Animals and the Guidelines of Mississippi State University. The animal care system at Mississippi State University is accredited by the American Association for Accreditation of Laboratory Animal Care, and the work described here adhered to the guidelines of that organization as well. The work was approved by the Mississippi State University Animal Care and Use Committee and animal care (protocol #07-066) was supervised by Dr. Lucy Senter, a board certified Laboratory Animal Veterinarian.

### Real time RT-PCR

Samples of RAW264.7 cells were lysed, RNA was purified with the RNeasy Plus Mini Kit 50 (# 74134, Qiagen, Valencia, CA). Cell lysates were homogenized with QiaShredders (# 79654, Qiagen). Samples were prepared with the SuperScript III Platinum SYBR Green One-Step qRT-PCR Kit with ROX reference dye (#11746-100, Invitrogen, Carsbad, CA). Real time RT-PCR was performed with the Stratagene Mx3005P QPCR System (Agilent Technologies, Cedar Creek, TX). Primers were designed with the Roche primer design software (Universal Probe Library, Primer 3 settings, Roche Applied Sciences, Indianapolis, IN, USA).

Mouse TNF-α:

forward: 5′-TGCCTATGTCTCAGCCTCTTC-3′

reverse: 5′-GAGGCCATTTGGGAACTTCT-3′

Mouse 18S:

forward: 5′-AAATCAGTTATGGTTCCTTTGGTC-3′

reverse: 5′-GCTCTAGAATTACCACAGTTATCCAA-3′

All samples were normalized to 18S. Data were analyzed using the ΔΔCt method.

### TACE inhibitor experiment

Cultures of RAW264.7 cells were treated with ethanol, LPS or poly I:C as described above. In the initial experiments with TACE inhibition, 1 mg TAPI-0 was reconstituted in 20 µl DMSO to a final concentration of 5 mg/ml, as recommended. Appropriate wells were treated with 25 µg/ml TAPI-0 or 5 µl/ml DMSO right before addition of LPS or poly I:C. Supernatants were harvested and further analyzed by mouse TNF-α ELISA. In later experiments, TAPI-0 was dissolved directly in complete medium to double the final concentration needed and added to each culture to dilute it to the final concentration, 20 µg/ml.

### Analysis of cell surface TNF-α by flow cytometry

FACS buffer at pH 7.2–7.4 was prepared with sterile phosphate buffered saline (Sigma Chemical Co., St. Louis, MO), 0.5% sodium azide, and 2% fetal bovine serum. In some cultures TAPI-0 was added, as noted above. The RAW264.7 cells were grown in 75 cm^2^ tissue culture flasks and transferred into sterile, endotoxin free Eppendorf tubes at 2.5×10^6^/ml. Appropriate tubes were treated with 86.8 mM EtOH for 30 min, and with 20 µg/ml TAPI-0, 100 ng/ml LPS, or 50 µg/ml poly I:C for another 2 h. The caps were left open and loosely covered with sterile lids to ensure air supply to the cells. Then, cells were pelleted at 1500 rpm at 4°C for 4 min and re-suspended in 200 µl FACS buffer. Cells were then transferred into a 96 well V-bottom plate, pelleted and resuspended in 200 µl FACS buffer. Appropriate wells were labeled with 1 µl fluorochrome conjugated primary antibody and incubated for 20 min on ice. Antibodies were obtained from Invitrogen: Monoclonal rat anti mouse TNF-α FITC conjugate (# RM9011), and rat IgG FITC isotype control (# R101). Samples were washed twice and resuspended in 200 µl FACS buffer after final wash. Experiments were carried out on a BD FACS Calibur flow cytometry system (BD Biosciences, San Jose, CA, USA).

### Statistical analysis

Real time RT-PCR results were analyzed with the delta delta CT method, using Microsoft Excel 2004 for Mac, version 11.0 (Microsoft Corporation, Redmond, Wa, USA). For the NF-κB reporter study, the unpaired t-test was used to compare the LPS-treated group and the group treated with LPS plus ethanol. Although one untreated control animal was included, this was not included in the statistical analysis, because it was included only to confirm that the instrument settings were appropriate and were not yielding substantial emissions in the absence of treatment. For all experiments with more than two groups, results were analyzed by one way analysis of variance with the Newman-Keuls multiple comparison test to compare the significance of the difference of each mean value to every other mean value. A P value<0.05 was considered significant. Prism 4.0 software (GraphPad Software, INC, La Jolla, CA, USA) was used for analysis.
